# A deep learning approach for automatic tumor delineation in stereotactic radiotherapy for non-small cell lung cancer using diagnostic PET-CT and planning CT

**DOI:** 10.3389/fonc.2023.1235461

**Published:** 2023-08-04

**Authors:** Xuyao Yu, Lian He, Yuwen Wang, Yang Dong, Yongchun Song, Zhiyong Yuan, Ziye Yan, Wei Wang

**Affiliations:** ^1^ Department of Radiation Oncology, Tianjin Medical University Cancer Institute and Hospital, National Clinical Research Center for Cancer, Tianjin’s Clinical Research Center for Cancer, Key Laboratory of Cancer Prevention and Therapy, Tianjin, China; ^2^ Tianjin Medical University, Tianjin, China; ^3^ Perception Vision Medical Technologies Co Ltd, Guangzhou, China; ^4^ Department of Radiotherapy, Tianjin Cancer Hospital Airport Hospital, Tianjin, China

**Keywords:** deep learning approach, dual-modality segmentation, automatic tumor delineation, stereotactic radiotherapy, non-small-cell lung cancer (NLSCLC)

## Abstract

**Introduction:**

Accurate delineation of tumor targets is crucial for stereotactic body radiation therapy (SBRT) for non-small cell lung cancer (NSCLC). This study aims to develop a deep learning-based segmentation approach to accurately and efficiently delineate NSCLC targets using diagnostic PET-CT and SBRT planning CT (pCT).

**Methods:**

The diagnostic PET was registered to pCT using the transform matrix from registering diagnostic CT to the pCT. We proposed a 3D-UNet-based segmentation method to segment NSCLC tumor targets on dual-modality PET-pCT images. This network contained squeeze-and-excitation and Residual blocks in each convolutional block to perform dynamic channel-wise feature recalibration. Furthermore, up-sampling paths were added to supplement low-resolution features to the model and also to compute the overall loss function. The dice similarity coefficient (*DSC*), precision, recall, and the average symmetric surface distances were used to assess the performance of the proposed approach on 86 pairs of diagnostic PET and pCT images. The proposed model using dual-modality images was compared with both conventional 3D-UNet architecture and single-modality image input.

**Results:**

The average *DSC* of the proposed model with both PET and pCT images was 0.844, compared to 0.795 and 0.827, when using 3D-UNet and nnUnet. It also outperformed using either pCT or PET alone with the same network, which had *DSC* of 0.823 and 0.732, respectively.

**Discussion:**

Therefore, our proposed segmentation approach is able to outperform the current 3D-UNet network with diagnostic PET and pCT images. The integration of two image modalities helps improve segmentation accuracy.

## Introduction

Lung cancer is a leading cause of global cancer incidence and mortality (1). Stereotactic body radiation therapy (SBRT) has been recommended by the European Society for Medical Oncology and the National Comprehensive Cancer Network as a standard clinical treatment for patients with non-small cell lung cancer (NSCLC) who cannot undergo surgical resection and has negative lymph nodes (2). Accurate delineation of the target area is crucial to ensure high-dose irradiation of the lesion while minimizing the dose to normal lung tissue (3). This process is difficult due to the distortion of normal structures caused by pneumonia, atelectasis, and pulmonary fibrosis in lung cancer patients. For adequate tumor tissue visualization and staging, multi-modality ^18^F-fluorodeoxyglucose (FDG) positron emission tomography (PET) and computed tomography (CT) images are emerging as important oncologic imaging techniques (4, 5). PET-CT combines the sensitivity of PET in detecting areas of abnormal function and anatomical localization from CT. Tumors usually exhibit higher FDG uptake than surrounding normal tissues in PET images. However, the low spatial resolution cannot accurately determine the spatial extent. CT provides detailed anatomical information with high resolution. However, CT has limited physiological information, and sometimes, there is a similar contrast between the tumor and surrounding soft tissue (4, 6, 7). It is essential to enrich standard anatomical imaging, i.e., CT, with the information on tumor biology gained by PET to better select and delineate SBRT target volumes.

With the steady increase in clinical indications for PET-CT imaging, the delineation of target volumes in radiotherapy planning relies more on PET and CT images for complementary information. However, in many cases, this is still a manual process by the oncologist on a slice-by-slice basis, with limited support from automated techniques. This can be labor-intensive, time-consuming, and prone to errors and inconsistency (8).

PET-CT images have a great impact on tumor visualization and staging; however, an integrated PET-CT system has not become the standard of care in SBRT planning for NSCLC. Until recently, anatomical imaging with CT or MRI scan was the only information available in the treatment planning process in many sites (9). Several automatic delineation methods on PET-CT have been reported for NSCLC (6, 10–13), but all methods work on images from an integrated PET-CT scanner, which has been registered through the hardware in the scanner. Song et al. designed an adaptive context term for the objective function to achieve consistent segmentation results between PET and CT (14). Ju et al. used a random walk method as an initial preprocessor to obtain object seeds, and a graph cut method was then used for lung tumor segmentation on PET-CT images (11). Li et al. proposed a two-stage segmentation approach, in which a fully convolutional network (FCN) was first used to generate a rough segmentation based on CT, and then a fuzzy variational model was utilized for the accurate segment on PET images and the input from the first stage (15). Zhong et al. adopted FCN co-segmentation for NSCLC in PET-CT images with two independent contours based on PET and CT images (10). In addition to lung cancer, for other cancer diseases, convolutional neural network (CNN)-based segmentation methods using co-registered PET-CT images have also been reported (16, 17). There are few works investigating the capability of automatic delineation based on diagnostic PET-CT and planning CT (pCT), which offers tumor metabolic information to CT with existing medical imaging resources. Note that when used for SBRT planning, no surgery or noticeable weight change should occur between the two scan times.

In this study, we propose a scheme for the automatic segmentation of the gross tumor volume (GTV) in NSCLC patients who undergo SBRT based on diagnostic PET-CT and pCT. The first step involves registering diagnostic CT taken with PET to pCT, and a transform matrix is obtained that permits directly registering PET to pCT. The 3D-UNet network is adopted as the backbone and supplemented with residual blocks, squeeze-and-excitation (SE) blocks (18), and auxiliary up-sampling paths to form the CNN-based segmentation model. The features from all levels of the network are merged into a single probability map as output. A comparison with dual-modality 3D-UNet is carried out to demonstrate the efficacy of our model. A single-modality image set from either PET or CT is used as the input to the proposed network to evaluate the contribution of each imaging modality and illustrate the superiority of using both modalities in the segmentation task. In total, the research suggests that the combined diagnostic PET-CT and pCT dual-modality segmentation approach should enable improved GTV segmentation accuracy for SBRT planning.

## Materials and methods

A total of 86 lung cancer patients who received SBRT were analyzed in this study following institutional review board approval. All patients had CT images (CT scanner; Philips Brilliance Big Bore CT, Amsterdam, Netherlands) for simulation and PET-CT images (PET-CT scanner; Discovery MI, GE Healthcare, Milwaukee, WI, USA) taken within 1 month (14.8 ± 10.3 days) before the simulation CT scans. The data collection spanned from January 2012 to January 2022. According to the GTV manual delineation results, the tumor size ranged from 0.33 to 57.90 cm^3^ with a mean volume of 12.15 ± 11.99 cm^3^. For all recruited patients, no surgery or treatment occurred between the two scans, so the tumor volume was considered the same. The fused PET-CT images were printed out as guidance when delineating the GTV on the pCT images. All contouring was completed using Precision (version 1.1.1.1; Accuray, Sunnyvale, CA, USA). In this study, while physicians referred to the fused PET-CT images to define the GTV contours on pCT images, the PET images and pCT images were not fused since they were not registered.

### Overview of the segmentation workflow

**Figure 1:**
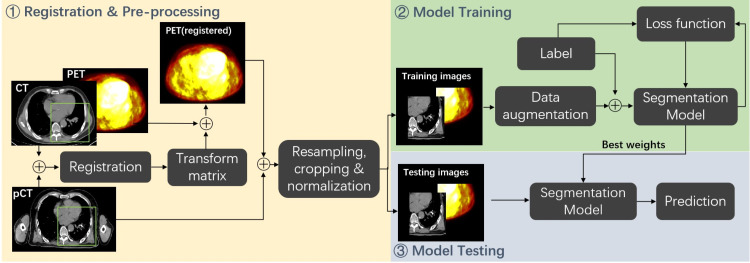
Overview of the segmentation workflow.

**Figure 1** illustrates the segmentation framework used in our study. The workflow consists of three main stages. In the first stage, the diagnostic CT images taken with the PET images are registered around the tumor region to the pCT images to generate the transform matrix, which allows for direct registering PET images to the pCT images. Then, patches are generated with the image pre-processing step, which includes resampling, cropping, and normalization. In the second stage, a 3D-UNet-based architecture with residual layers, SE normalization (SE Norm), and auxiliary paths is trained with PET-CT images to segment the GTV region. In the last stage, the weights from the best-trained model are selected to generate GTV contours on the testing set. The contours are compared with the manual delineation results to calculate the evaluation metrics.

### pCT and PET image registration

The registration is performed with the 3D Slicer 5.0.2 software landmark registration module (https://www.slicer.org/). For each data set, 8 to 10 pairs of landmarks in diagnostic CT and pCT are manually selected for rigid registration by an experienced clinician. In this process, the pCT image set is considered the fixed image, and the CT image set is the moving image. Note that only the volume centered around the tumor region (≥150 × 150 × 150 mm^3^) is considered as registration volume since the respiratory motion and patient positioning difference between two scan times make registration of the whole lung volume challenging. If multiple targets exist, a larger volume should be registered. A transform matrix from rigid registration is then generated and exported from the 3D Slicer and used to translate and rotate the PET image arrays. This process is automated by a Python script (Python 3.7). To this end, PET and pCT images are believed to be registered given the diagnostic PET and CT images are already registered by the scanner.

### Image pre-processing

Once pCT and PET images are registered, they are first resampled to a common resolution of 1 × 1 × 1 mm^3^ with trilinear interpolation. The PET and pCT images are then cropped to a patch of 144 × 144 × 144 voxels to ensure the lesion is included in the 3D volume. To remove the unrelated image details, the pCT intensities are clipped in the range of [−800, 800] Hounsfield Units and then mapped to [−1, 1]. The PET images are transformed independently with Z-score normalization for each patch.

### Network architecture

**Figure 2:**
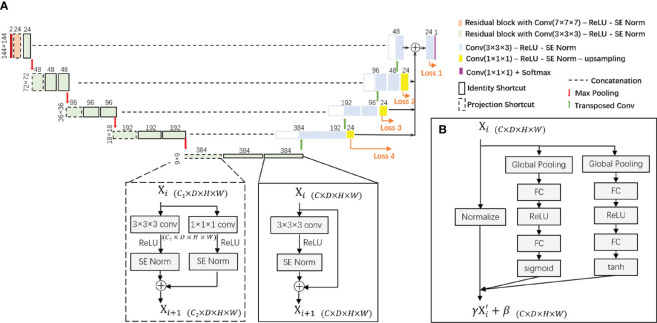
**(A)** The proposed 3D-UNet-based network architecture with SE Norm layer and residual layer with identity and projection shortcuts. **(B)** The detailed structure of the SE Norm layer. SE Norm, squeeze-and-excitation normalization.

The proposed segmentation model is built upon a 3D-UNet architecture as illustrated in **Figure 2**. This is a single-path model where PET and pCT are concatenated into a single matrix for input. The basic module of the encoder and decoder was a convolutional block with rectified linear unit (ReLU) activation function and SE Norm. The operation of down-sampling in the encoder is achieved by max pooling with a 2 × 2 × 2 kernel size. Residual blocks with convolutional blocks, SE Norm blocks, and shortcut connections (**Figure 2**) are employed in the encoder. The up-sampling operation in the decoder is achieved by using a 3 × 3 × 3 transposed convolution. Initially, 24 feature maps are extracted in this network, which are doubled along each down-sampling operation in the encoder. The number of feature maps is halved by each transposed convolution in the decoder. At the end of the decoder, the spatial size achieved is the same as the initial input size. A 1 × 1 × 1 convolutional block is applied with a softmax classifier to generate a voxel-level probability map and the final prediction.

In the decoder, three up-sampling paths are used to transfer low-resolution features by applying a 1 × 1 × 1 convolutional block to reduce the number of channels and utilizing trilinear interpolation to increase the spatial size of the feature maps. We also obtain label probability maps from the three paths and compute the weighted sum of all loss functions to the overall loss function. For each loss function, the sum of Dice loss and focal loss is used: 


(1) $$\text{L}=L_{1}+\sum_{i=2}^{4}w_{i}\cdot L_{i},$$

where *L_i_* = *L_Dice_* + *L_Focal_* is the loss function from each layer in the network architecture and *w_i_* is the weight for each layer.

### Network training

Of the total 86 pairs of PET-CT scans, 54 pairs were used as the training set, 18 pairs as the validation set, and 14 pairs as the testing set. The selection procedure began by sorting all scan pairs according to tumor volume (GTV voxel number). The whole patient group was divided into 18 groups with group 1 patients having the smallest GTV volumes and group 18 having the largest GTV volumes. Three scan pairs in each group were selected to be included in the training set, and the rest of the patients were randomly assigned to validation and testing sets. This stratified strategy ensured that the training set is representative of the whole data set in terms of tumor volume. All parameters were tuned on the training set. All reported results were obtained on the test set. The best model on the validation set is selected and used to evaluate the performance of the testing set.

Several data augmentation methods were adopted to enhance the training set. For each pair of co-registered PET-CT scans in the training set, rotation from 0 to 45 flip operation and random rotation were performed to obtain additional training sets.

### Compared methods

Experiments were designed to evaluate the performance of the proposed model and the contribution of dual-modality input. We compared the performance of our proposed model with 3D-UNet and nnUNet as baseline models (19, 20) using both PET and pCT as input. The single-modality data set of either PET or pCT was then used as input to the proposed model. Those experiments were carried out with the same data set from 86 patients and the same hyper-parameters for the network, and the results were compared with those of our proposed model. For nnUNet, a 3D full-resolution UNet configuration was selected, and registered pCT and PET images were cropped to patches of 144 × 144 × 144 mm^3^ for the network.

The segmentation accuracy of the proposed method and other comparison algorithms was evaluated by the following criteria:

1. Dice similarity coefficient (*DSC*), which measures the volume overlap of two segmentations (21, 22):


(2) $$DSC=\;\frac{2\left|A\cap^{}B\right|}{\left|A\right|\cup^{}\left|B\right|}$$

where *A* and *B* denote the manual and automatic segmentation results, respectively. Therefore, a higher *DSC* indicates a more precise segmentation performance.

2. Recall and precision scores, which show the true positive rate and positive prediction value:


(3) $$\text{recall}=\;\frac{TP}{TP+FN},$$

(4) $$\text{precision}=\;\frac{TP}{TP+FP},$$

where *TP*, *FP*, and *FN* are true positive, false positive, and false negative, respectively.

3. Average symmetric surface distance (*ASSD*), which is defined as follows:


(5) $$ASSD\left(\text{A},\text{B}\right)=\;\frac{\sum^{}a\in A^{min_{b\in B^{d\left(a,b\right)}}}+\sum^{}b\in B^{min_{a\in A^{d\left(a,b\right)}}}}{\left|S\left(A\right)\right|+\left|S\left(B\right)\right|},$$

where S() indicates the set of pixels on the surface, d() means Euclidean distance between two points, and | | represents the number of pixels in the set. Therefore, lower ASSD means a shorter distance between two surfaces.

Based on the abovementioned metrics, paired t-test is conducted (Python 3.9 scipy.stats package) to verify if there is a statistical difference between the proposed and comparison methods. A *p*-value smaller than 0.05 is considered significantly different. The training and inference time, as well as the GPU memory consumption for each method, were also quantified to better evaluate the performance of the proposed algorithm.

### Experiment setting

For the deep learning environment, we built Python with version 3.9.12 and PyTorch with version 1.11.0 on the Ubuntu 16.04.2 LTS. All networks ran on one Quadro P6000 GPU with 24 GB of memory. All models were trained by the Adam optimization method with a batch size of 1 for 600 epochs. The cosine annealing schedule was applied to reduce the learning rate from 1e−3 to 1e−6 within every 25 epochs.

## Results

**Table 1** shows the mean and standard deviation of the evaluation metrics, i.e., *DSC*, precision, recall, and ASSD, for the proposed and comparison models with single and dual image modalities as input. It was observed that the proposed model with dual image modalities has a superior or comparable performance in all evaluation metrics. When using two image modalities as input, the proposed model outperforms the 3D-UNet in all evaluation metrics. It also outperforms nnUNet for the majority of the metrics, though no significant difference was found. Moreover, there were significant differences in terms of *DSC* and *ASSD* between the proposed model and the 3D-UNet model (*p*-values were 0.011 and 0.021). This demonstrates that the proposed network has more advanced and perceptive learning ability than the conventional 3D-UNet. **Table 1** also shows higher segmentation accuracy based on dual image modalities than single modality using either pCT or PET alone. There are significant differences in all evaluation metrics between PET and dual image modalities as input (*p*-values are 0.0007, 0.035, 0.027, and 0.0003 for *DSC*, precision, recall, and *ASSD*, respectively), while no significant difference is observed between pCT and dual modalities.

**Table 1:** Statistics of our proposed method and the compared methods on the testing set.

Image	Model	DSC	Precision	Recall	ASSD
pCT	Proposed	0.823 ± 0.093	0.794 ± 0.153	0.877 ± 0.052	0.988 ± 0.557
PET	Proposed	0.732 ± 0.111	0.763 ± 0.153	0.749 ± 0.155	1.674 ± 0.691
pCT-PET	Proposed	0.844 ± 0.058	0.840 ± 0.100	0.859 ± 0.071	0.887 ± 0.384
pCT-PET	3D-UNet	0.795 ± 0.102	0.822 ± 0.158	0.798 ± 0.110	1.312 ± 0.618
pCT-PET	nnUNet	0.827 ± 0.082	0.863 ± 0.128	0.789 ± 0.045	0.994 ± 0.580

DSC, Dice similarity coefficient; ASSD, average symmetric surface distance; pCT, planning computed tomography; PET, positron emission tomography.

**Table 2** reports the training and inference time as well as the GPU memory usage during model training for all dual-modality segmentation methods. Compared with 3D-UNet, the training and inference time taken by the proposed model increased by 8% and 27%, respectively, while the GPU memory usage increased by 40%. This is not surprising since the scaling operation in the excitation stage in the SE Norm block generated a weighted channel vector with the same size as the input, which was subsequently applied element-wise to the input. This rendered the training process slower and more memory intensive. However, this additional time and space overhead were justified by its contribution to model performance. The nnUNet was trained with the fixed parameter for 1,000 epochs, while the proposed model was trained for 600 epochs in this study. For the average training time taken per epoch, the proposed model requires one-fourth of the time cost by nnUnet, which presents a similar ratio in inference time between the two models for each patient. This is due to the factor that the sliding window approach leads to a longer prediction time for nnUNet, which exists in both training, i.e., validation stage and inference.

**Table 2:** Time and memory usage comparison across dual-modality segmentation methods.

	3D-UNet	nnUNet	Proposed
**Training time (hour)**	9.3	66.8	10.0
**Inference time (sec per patient)**	1.5	7.9	1.9
**GPU memory usage (MB)**	8,969	8,801	12,543

**Figure 3:**
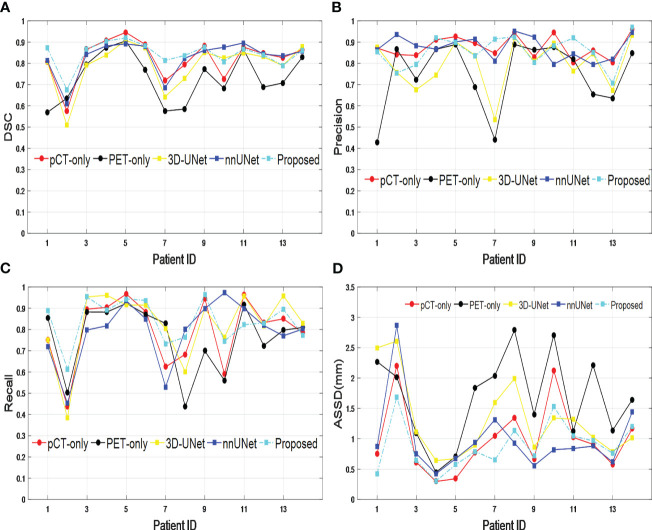
Comparison across different models and input image modalities from 14 test data sets based on **(A)** *DSC*, **(B)** precision, **(C)** recall, and **(D)** *ASSD*. DSC, Dice similarity coefficient; ASSD, average symmetric surface distance.

The performance of our proposed dual-modality segmentation method was also compared with that of other models and input image modalities for all 14 test data sets. **Figure 3** shows that our proposed segmentation model achieved overall improvement for all evaluation metrics and also gave the most stable segmentation performance in the entire test data set. The curves from the PET-only method show the highest fluctuations for all evaluation metrics, which suggests for some testing data the PET-only method has failed. However, the pCT-only method achieved performance very close to the dual image modality method, which demonstrates a big contribution to segmentation. When incorporating two image modalities as input, the proposed network architecture provides more accurate and consistent segmentation over the 3D-UNet and nnUNet models, which is due to the improved representational power from the SE block.

**Figure 4:**
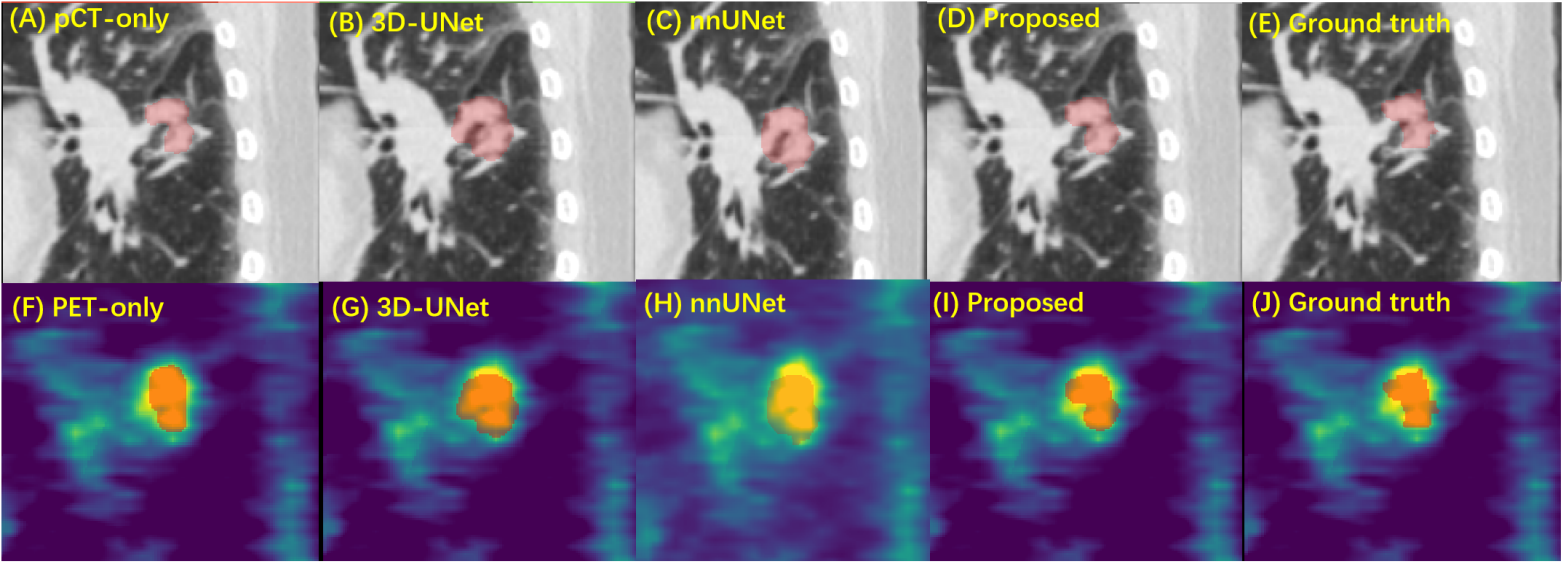
The performance of GTV segmentation by proposed model, compared with different input modalities and 3D-UNet model. **(A–E)** Segmentation results (red) on pCT images by pCT image only with the proposed model, dual-modality input with 3D-UNet model, nnUNet model, the proposed model, and the manual delineation. **(F–H)** Segmentation results (red) on PET images by PET image only with proposed model, dual-modality input with 3D-UNet model, nnUNet model, the proposed model, and the manual delineation. GTV, gross tumor volume; pCT, planning computed tomography; PET, positron emission tomography.

**Figure 4** shows a typical tumor delineation with different input image modalities and segmentation models on pCT and PET images. In this example, methods with SE Norm blocks and auxiliary paths, i.e., shown in **Figures 4A, D, F, I**, present closer boundaries to the manual delineation than 3D-UNet and nnUNet with dual-modality input. This indicates the proposed network is able to increase its sensitivity to informative features so that they can be exploited by subsequent transformations and suppress less useful ones. For the PET-only method, the blurry boundary is the main reason for the inadequate performance compared with the proposed method.

## Discussions

The precision of the GTV delineation is crucial for accurate treatment and dose assessment in lung cancer SBRT. Automated segmentation techniques, such as deep learning, have the potential to reduce intra- and inter-observer variabilities in manual GTV delineations and are in high demand in clinical SBRT practices. Jordan Wong et al. proposed a deep learning-based auto-segmentation model for lung stereotactic ablative radiotherapy, and the *DSC* and 95% Hausdorff distance (*HD*) of the GTV were 0.71 and 5.23 mm, respectively (23). Dense V-networks (DVNs) were used to develop a GTV automated segmentation for planning lung cancer SBRT, and 3D *DSC* and *HD* were 0.832 ± 0.074 and 4.57 ± 2.44 mm, respectively (24). PET-CT image modalities hold a special place for disease characterization since they contain complementary information about the metabolism and the anatomy of cancer (25). Therefore, PET-CT can be used to develop a dual-modality segmentation approach to the GTV for lung cancer SBRT. Several previous studies have shown that segmentation based on CT and PET images can improve accuracy in lung tumor segmentation compared with using single-modality images (10, 11, 14, 15). Many of the published works require two probability maps to be generated for each imaging modality, which results in extra clinical workflow. In this paper, we determine the tumor region effectively and accurately with only one probability map, which is estimated by taking advantage of both PET and pCT images. The novelty of this model lies in the SE blocks (18) to dynamically enhance the important feature and the up-sampling paths to supplement low-resolution features in the model. Additionally, the up-sampling paths are included in the overall loss function calculation to supervise the training of lower-layer parameters, which can further improve the discriminative capability of the network. The new components brought to the proposed model have significantly improved the accuracy and consistency for GTV segmentation in SBRT when comparing the evaluation metrics (**Table 1**, **Figure 3**) between the proposed model and 3D-UNet and nnUNet.

This study overcomes the weakness that the integrated PET-CT scanner is not routinely used in RT planning, while functional contrast from PET is always desired to provide adequate tumor visualization. Compared with previously published studies, it is more challenging to fuse diagnostic PET and pCT image features since the two sets of images may have different patient positions and breathing phases. Here, we use a semi-automatic approach for registration and ensure at least 150 × 150 × 150 mm^3^ around the tumor region to be well-registered, which requires an estimation of tumor location before registration. One recently published work also demonstrates the capability of segmenting lung tumors on diagnostic PET and pCT using a stratified method based on tumor volume [28]. Rather than knowing the tumor volume in advance, our model copes with tumors of varied sizes, which brings convenience to clinical deployment.

The DSC of our proposed network with dual image modalities is 0.844, which is better than the average values []. Evaluation metrics from the CT-only approach presented very close results to the dual-modality approach (*DSC*: 0.823 vs. 0.844), and no significant difference was found, which indicates the contribution from CT image in dual-modality segmentation is very remarkable. Regarding the PET-only approach, even though the contribution is small in this work, it still improves the segmentation. We notice that in some cases there are displacements between PET and CT images around the tumor region, which can be caused by imperfect PET image registration due to subject motion between PET and CT scans. This can be one reason that induces lower segmentation performance in the PET-only approach. Some studies reported a deep learning-based motion correction image registration model to solve this problem (26, 27), which is a future direction for us to improve the PET images’ contribution to segmentation.

Although the proposed network with dual image modalities has achieved improvement over the single-modality method, there is still much improvement needed in terms of performance and workflow. Manual registration between diagnostic PET and pCT is the most significant limitation for obtaining efficient GTV segmentation. Note that this limitation does not alter the paper’s findings and conclusion about segmentation accuracy. Previously published work suggests the capability of inter-modality image registration with a deep learning-based method (28, 29). Our promising approach is the use of a deep learning-based network to directly register diagnostic PET to pCT. The automatic delineation of the target area not only can reduce contouring time and decrease interobserver variability but also can improve dose consistency and accuracy. Future work should look into a dosimetric calculation based on auto-segmented and manually-segmented GTV contours to assess clinical validity.

## Conclusions

In this study, we proposed a novel dual-modality, 3D-UNet-based network for the segmentation of tumors in diagnostic PET and pCT images. The proposed neural network can make full use of the advantages from both modalities, i.e., the metabolic information from PET and anatomical information from pCT. The proposed neural network was validated on clinic images of 86 patients with lung cancer, including one set of PET-CT images and another set of pCT images. The results showed that the proposed network is effective and robust and achieved significant improvement over the original 3D-UNet model and is superior or comparable to nnUNet in most evaluation metrics when using two modalities. It also demonstrated that dual modality outperformed single-modality for automatic GTV delineation in SBRT.

## Data availability statement

The raw data supporting the conclusions of this article will be made available by the corresponding authors without undue reservation.

## Ethics statement

The studies involving human participants were reviewed and approved by Ethical Committee of Tianjin Medical University Cancer Institute and Hospital. The patients/participants provided their written informed consent to participate in this study. Written informed consent was obtained from the individual(s) for the publication of any potentially identifiable images or data included in this article.

## Author contributions

Design of the study: XYY, LH, and WW. Acquisition of data: YWW, YD, YCS, and ZYYu. Analysis and interpretation of data: LH and XYY. Drafting and revision of the article: LH, XYY, WW, and ZYYa. Review and approval of the manuscript: all. All authors contributed to the article and approved the submitted version.

## References

[1] Thandra KC Barsouk A Saginala K Padala SA Barsouk A Rawla P. Epidemiology of Non-Hodgkin’s lymphoma. Med Sci (Basel) (2021) 9(1):5. doi: 10.3390/medsci9010005 33573146 PMC7930980

[2] Wood DE Kazerooni EA Aberle D Berman A Brown LM Eapen GA. NCCN guidelines^®^ Insights: lung cancer screening, version 1.2022. J Natl Compr Canc Netw (2022) 20(7):754–64. doi: 10.6004/jnccn.2022.0036 35830884

[3] Green OL Henke LE Hugo GD. Practical clinical workflows for online and offline adaptive radiation therapy. Semin Radiat Oncol (2019) 29(3):219–27. doi: 10.1016/j.semradonc.2019.02.004 PMC6487881 31027639

[4] Vaz SC Adam JA Delgado Bolton RC Vera P van Elmpt W Herrmann K. Joint EANM/SNMMI/ESTRO practice recommendations for the use of 2-[18F]FDG PET/CT external beam radiation treatment planning in lung cancer V1.0. Eur J Nucl Med Mol Imaging (2022) 49(4):1386–406. doi: 10.1007/s00259-021-05624-5 PMC8921015 35022844

[5] Fonti R Conson M Del Vecchio S. PET/CT in radiation oncology. Semin Oncol (2019) 46(3):202–9. doi: 10.1053/j.seminoncol.2019.07.001 31378377

[6] Kumar A Fulham M Feng D Kim J. Co-learning feature fusion maps from PET-CT images of lung cancer. IEEE Trans Med Imaging (2019) 39(1):31217099. doi: 10.1109/TMI.2019.2923601 31217099

[7] van Baardwijk A Bosmans G Boersma L Buijsen J Wanders S Hochstenbag M. PET-CT-based auto-contouring in non-small-cell lung cancer correlates with pathology and reduces interobserver variability in the delineation of the primary tumor and involved nodal volumes. Int J Radiat Oncol Biol Phys (2007) 68(3):771–8. doi: 10.1016/j.ijrobp.2006.12.067 17398018

[8] Devic S Tomic N Faria S Menard S Lisbona R Lehnert S. Defining radiotherapy target volumes using 18F-fluoro-deoxy-glucose positron emission tomography/computed tomography: still a Pandora’s box? Int J Radiat Oncol Biol Phys (2010) 78(5):1555–62. doi: 10.1016/j.ijrobp.2010.02.015 20646840

[9] Jelercic S Rajer M. The role of PET-CT in radiotherapy planning of solid tumours. Radiol Oncol (2015) 49(1):1–9. doi: 10.2478/raon-2013-0071 25810695 PMC4362600

[10] Zhong Z Kim Y Plichta K Allen BG Zhou L Buatti J. Simultaneous cosegmentation of tumors in PET-CT images using deep fully convolutional networks. Med Phys (2019) 46(2):619–33. doi: 10.1002/mp.13331 PMC6527327 30537103

[11] Ju W Xiang D Zhang B Wang L Kopriva I Chen X. Random walk and graph cut for co-segmentation of lung tumor on PET-CT images. IEEE Trans Image Process. (2015) 24(12):5854–67. doi: 10.1109/TIP.2015.2488902 26462198

[12] Kao Y-S Yang JJC. Deep learning-based auto-segmentation of lung tumor Pet/Ct scans: A systematic review. Clin Trans Imaging (2022) 10(2):217–23. doi: 10.1007/s40336-022-00482-z

[13] Fu X Bi L Kumar A Fulham M Kim J. Multimodal spatial attention module for targeting multimodal PET-CT lung tumor segmentation. IEEE J BioMed Health Inform (2021) 25(9):3507–16. doi: 10.1109/JBHI.2021.3059453 33591922

[14] Song Q Bai J Han D Bhatia S Sun W Rockey W. Optimal co-segmentation of tumor in PET-CT images with context information. IEEE Trans Med Imaging (2013) 32(9):1685–97. doi: 10.1109/TMI.2013.2263388 PMC3965345 23693127

[15] Li L Zhao X Lu W Tan S. Deep learning for variational multimodality tumor segmentation in PET/CT. Neurocomputing (2020) 392:277–95. doi: 10.1016/j.neucom.2018.10.099 PMC7405839 32773965

[16] Iantsen A Visvikis D Hatt M. (2021). Squeeze-and-excitation normalization for automated delineation of head and neck primary tumors in combined Pet and Ct images, in: Head and Neck Tumor Segmentation: First Challenge, HECKTOR 2020, Held in Conjunction with MICCAI 2020, Lima, Peru, October 4, 2020. Springer, *Proceedings 1*.

[17] Zhao L Lu Z Jiang J Zhou Y Wu Y Feng Q. Automatic nasopharyngeal carcinoma segmentation using fully convolutional networks with auxiliary paths on dual-modality PET-CT images. J Digit Imaging (2019) 32(3):462–70. doi: 10.1007/s10278-018-00173-0 PMC6499852 30719587

[18] Hu J Shen L Albanie S Sun G Wu E. Squeeze-and-excitation networks. IEEE Trans Pattern Anal Mach Intell (2020) 42(8):2011–23. doi: 10.1109/TPAMI.2019.2913372 31034408

[19] Çiçek Ö Abdulkadir A Lienkamp SS Brox T Ronneberger O. (2016). 3d U-net: learning dense volumetric segmentation from sparse annotation, in: Medical Image Computing and Computer-Assisted Intervention–MICCAI 2016: 19th International Conference, Athens, Greece, October 17-21, 2016. Springer, *Proceedings, Part II 19*.

[20] Isensee F Jaeger PF Kohl SA Petersen J Maier-Hein KH. nnU-Net: a self-configuring method for deep learning-based biomedical image segmentation. Nat Methods (2021) 18(2):203–11. doi: 10.1038/s41592-020-01008-z 33288961

[21] Yao AD Cheng DL Pan I Kitamura F. Deep learning in neuroradiology: A systematic review of current algorithms and approaches for the new wave of imaging technology. Radiol Artif Intell (2020) 2(2):e190026. doi: 10.1148/ryai.2020190026 33937816 PMC8017426

[22] Zou KH Warfield SK Bharatha A Tempany CM Kaus MR Haker SJ. Statistical validation of image segmentation quality based on a spatial overlap index. Acad Radiol (2004) 11(2):178–89. doi: 10.1016/s1076-6332(03)00671-8 PMC1415224 14974593

[23] Wong J Huang V Giambattista JA Teke T Kolbeck C Giambattista J. Training and validation of deep learning-based auto-segmentation models for lung stereotactic ablative radiotherapy using retrospective radiotherapy planning contours. Front Oncol (2021) 11:626499. doi: 10.3389/fonc.2021.626499 34164335 PMC8215371

[24] Cui Y Arimura H Nakano R Yoshitake T Shioyama Y Yabuuchi H. Automated approach for segmenting gross tumor volumes for lung cancer stereotactic body radiation therapy using CT-based dense V-networks. J Radiat Res (2021) 62(2):346–55. doi: 10.1093/jrr/rraa132 PMC7948852 33480438

[25] Oreiller V Andrearczyk V Jreige M Boughdad S Elhalawani H Castelli J. Head and neck tumor segmentation in PET/CT: The HECKTOR challenge. Med Image Anal (2022) 77:102336. doi: 10.1016/j.media.2021.102336 35016077

[26] Li T Zhang M Qi W Asma E Qi J. Motion correction of respiratory-gated PET images using deep learning based image registration framework. Phys Med Biol (2020) 65(15):155003. doi: 10.1088/1361-6560/ab8688 32244230 PMC7446936

[27] Xiao H Teng X Liu C Li T Ren G Yang R. A review of deep learning-based three-dimensional medical image registration methods. Quant Imaging Med Surg (2021) 11(12):4895–916. doi: 10.21037/qims-21-175 PMC8611468 34888197

[28] Cao X Yang J Wang L Xue Z Wang Q Shen D. Deep learning based inter-modality image registration supervised by intra-modality similarity. Mach Learn Med Imaging (2018) 11046:55–63. doi: 10.1007/978-3-030-00919-9_7 31098597 PMC6516490

[29] Chen X Diaz-Pinto A Ravikumar N Frangi AF. Deep learning in medical image registration. Prog Biomed Eng (2021) 3(1):012003. doi: 10.1088/2516-1091/abd37c

